# Developing a Novel Two‐Dimensional Culture System to Enrich Human Prostate Luminal Progenitors that Can Function as a Cell of Origin for Prostate Cancer

**DOI:** 10.5966/sctm.2016-0243

**Published:** 2016-09-29

**Authors:** Dingxiao Zhang, Kevin Lin, Yue Lu, Kiera Rycaj, Yi Zhong, Hsueh‐Ping Chao, Tammy Calhoun‐Davis, Jianjun Shen, Dean G. Tang

**Affiliations:** ^1^ Department of Epigenetics and Molecular Carcinogenesis, University of Texas, MD Anderson Cancer Center, Houston, Texas, USA; ^2^ Department of Pharmacology and Therapeutics, Roswell Park Cancer Institute, Buffalo, New York, USA; ^3^ Center for Cancer Epigenetics, University of Texas MD Anderson Cancer Center, Houston, Texas, USA; ^4^ Center for Stem Cell and Developmental Biology, University of Texas MD Anderson Cancer Center, Houston, Texas, USA; ^5^ Center for RNA Interference and Non‐Coding RNAs, University of Texas MD Anderson Cancer Center, Houston, Texas, USA; ^6^ Center for Molecular Carcinogenesis, University of Texas MD Anderson Cancer Center, Houston, Texas, USA; ^7^ Cancer Stem Cell Institute, Research Center for Translational Medicine, East Hospital, Tongji University, Shanghai, People’s Republic of China

**Keywords:** Prostate epithelial progenitor cells, Stem cells, Cancer cell of origin, Prostate cancer

## Abstract

Elucidating the cell of origin of cancer has great significance in stratifying patients into appropriate treatment groups and for developing novel targeted therapies. Early studies demonstrate that only stem‐like basal cells in the normal human prostate (NHP) can function as the cell of origin for prostate cancer (PCa). Here, we show that the organoids derived from bulk NHP luminal cells can also be tumorigenically transformed. We further show that the WIT medium, which is used to culture human mammary epithelial progenitor cells, when combined with the ROCK inhibitor, can readily propagate a population of progenitor‐like cells from the primary NHP luminal cell isolates. Such functionally defined luminal progenitors can be transformed by distinct sets of genetic perturbations (i.e., AR+AKT/ERG or c‐MYC+PTEN knockout) to form tumor glands. Genome‐wide RNA‐Seq analysis of freshly purified unperturbed human benign prostatic basal and luminal cells and culture‐expanded lineage‐specific stem/progenitor populations reveals that the luminal progenitors possess a distinct gene expression profile that is greatly enriched in advanced, castration‐resistant, and metastatic PCa, and it associates with poor patient survival. The ability of the simple two‐dimensional culture system reported herein to greatly enrich NHP progenitor‐like cells should facilitate biological and biochemical studies as well as high‐throughput screening in these cells and in progenitor‐like PCa cells. Stem Cells Translational Medicine *2017;6:748–760*

**Significance Statement**

The development of a novel two‐dimensional cell culture system demonstrated that the luminal progenitor cells in adult human prostate can serve as a cell of origin for prostate cancer.

## Introduction

The prostate is an exocrine gland consisting of luminal, basal, and rare neuroendocrine (NE) cells [1]. Developmentally, the murine prostate originates from an ancestral p63^+^AR^−^ basal stem cell (SC) population [2]. Prostate regeneration assays demonstrate that SCs with the capacity to differentiate into all three cell types are localized to the basal layer of the mouse prostate [3]. Lineage‐tracing studies indicate that both basal and luminal cell layers in adult murine prostate contain lineage‐restricted stem/progenitor cells with more primitive SCs residing in the basal layer [4, 5]. In the human prostate, in vitro SC‐related and tissue regeneration (TR) assays demonstrate that the basal layer harbors regenerative SCs [6], whereas the luminal layer has multipotent progenitor cells [7].

The prostate is highly susceptible to tumorigenesis. Prostate cancer (PCa), most of which presents as adenocarcinomas and manifests a luminal cell phenotype, is a heterogeneous malignancy harboring functionally diverse subpopulations of cancer cells [8, 9]. Studies using genetic mouse models show that PCa can originate from both the basal and luminal lineages with luminal cells being the preferred transformation targets [4, 10]. However, earlier TR‐based assays have identified only a subset of basal stem‐like cells (i.e., CD49f^hi^Trop2^+^) that can function as the cell of origin for human PCa [6]. Although it is presently unclear what might account for the discrepancies in these two lines of studies, it is conceivable that bulk luminal cells defined by CD49f^lo^Trop2^+^ [6] may fail in the TR assays because the majority of cells in this population are terminally differentiated cells with very limited regenerative activity [4, 11] and the success of TR assays typically depends on stem/progenitor cell‐related properties. A recent study using three‐dimensional (3D) sphere cultures has provided evidence for a small population (<2%) of functionally defined luminal progenitor cells in the human prostate [7]. Very recently, Park et al. [12] showed that freshly purified CD26^+^ human prostate luminal cells could be transformed by lentivirus expressing c‐MYC and activated AKT1 in the 3D culture conditions to initiate PCa. However, in this study it was unclear which subset(s) of the bulk luminal population became transformed (e.g., bulk differentiated cells, rare luminal progenitors, or other type of luminal cells that gain SC properties because of overexpression of these oncogenes). Moreover, the putative prostatic luminal progenitors are very rare (<2% in the 3D organoids), making them difficult for biological/biochemical studies and for drug screening.

In this study, we demonstrated that freshly purified CD49f^lo^Trop2^+^ luminal cells, like the CD49f^hi^Trop2^+^ basal/stem cells, can be transformed by distinct sets of genetic elements to form histologically abnormal glandular structures resembling human PCa. We adopted and modified a culture system (i.e., WIT, a serum‐free defined medium originally optimized for the robust culture of human primary mammary luminal progenitor cells [13]) to greatly enrich functional human prostatic luminal progenitors that can regenerate prostatic glands and can be tumorigenically transformed to generate prostate tumors in vivo.

## Materials and Methods

### Human Primary Prostate Tissue Processing, Fluorescence‐Activated Cell Sorting, and Cell Culture

Primary benign prostate tissues were obtained from human PCa (HPCa) patients (supplemental online Table 1) undergoing radical prostatectomy with written informed patient consent (institutional review board approval no. LAB04‐0498). The HPCa processing protocol was described previously [11, 14]. The final dissociated single‐cell suspension was stained with PE‐CD49f, FITC‐CD26 (eBioscience, San Diego, CA, http://www.ebioscience.com), and APC‐Trop2 (R&D Systems, Minneapolis, MN, https://www.rndsystems.com) to separate basal (Trop2^+^CD49f^hi^ or CD49f^+^CD26^−^) and luminal (Trop2^+^CD49f^lo^ or CD49f^−^CD26^+^) populations. Cells were cultured on PureCol (Advanced BioMatrix, Carlsbad, CA, https://www.advancedbiomatrix.com) precoated dishes in prostate epithelial cell growth medium (PrEGM; Lonza, Basel, Switzerland, http://www.lonza.com) or optimized WIT medium for primary mammary epithelial cells (WIT‐P, catalog no. 00‐0045‐500; Stemgent, Cambridge, MA, https://www.stemgent.com) medium supplemented with 10 μM of p160ROCK inhibitor Y‐27632 dihydrochloride (Selleckchem, Houston, TX, http://www.selleckchem.com) to inhibit anoikis. The 3D organoid culture system was used to enrich stem/progenitor populations from both lineages [7]. Detailed materials and methods are described in the supplemental online data.

### Lentiviral Infections

Basic lentiviral procedures were previously described [9]. Lentivirus was produced in 293FT packaging cells. For PSAP‐GFP [9], AR‐GFP, and AKT/ERG‐RFP [6] lentiviruses, the titers were determined using GFP or RFP positivity in 293FT cells. Two MISSION shRNAs (TRCN0000002749 and TRCN0000002747; Sigma‐Aldrich, St. Louis, MO, http://www.sigmaaldrich.com) were used to knock down PTEN. For PTEN‐shRNAs, pCDH‐puro‐cMYC [15] and lentiCRISPR‐puro [16], the titers were roughly calculated based on the puromycin resistance (1 μg/ml) in 293FT cells. We usually infected the human prostate primary cell cultures at a multiplicity of infection (MOI) of 10 twice at day 1 and day 3 with a medium change at day 2 and then harvested cells for experiments 48–72 hours postinfection. Cells were trypsinized, counted, and seeded in 6‐well plates for colony formation and in 12‐well plates for sphere formation assays, or combined with mouse urogenital sinus mesenchyme (mUGSM) for tissue regeneration assays.

### RNA‐Seq

Basic procedures for RNA‐Seq have recently been published [11]. Dissociated bulk prostate cells or purified basal and luminal cell populations from HPCa167N benign tissues were short‐term expanded in either PrEGM or WIT for 7 to 9 days, followed by total RNA extraction using an RNeasy mini kit (Qiagen, Hilden, Germany, https://www.qiagen.com). cDNA libraries were constructed using the TruSeq Stranded Total RNA Preparation Kit (catalog no. RS‐122‐2301Illumina, San Diego, CA, http://www.illumina.com), which contained Ribo‐ZeroTM Gold to deplete rRNA. We amplified our libraries for only 10 polymerase chain reaction (PCR) cycles (instead of 15 suggested by manufacturer) to minimize amplification‐induced noise. Purified libraries were quantified using a Kapa library quantification kit (KAPA Biosystems, Wilmington, MA, https://www.kapabiosystems.com) and then loaded onto a cBot (Illumina) at a final concentration of 10 pm to perform cluster generation, followed by 2 × 76 bp sequencing on a HiSEquation 2500 (Illumina). Two libraries were pooled and loaded, producing an average of 400 M reads per lane. From each sample, we obtained approximately 100 M pairs of reads (200 M reads), indicating the high depth of sequencing.

### RNA‐Seq Data Processing and Bioinformatics

We mapped the sequencing reads to the reference human genome sequence (hg38) using TopHat (version 2.0.10) [17] and Bowtie 2 (version 2.1.0) [18]. The number of fragments in each known gene from GENCODE Release 21 [19] was enumerated using htseq‐count from HTSeq package (version 0.6.0) [20]. Genes with fewer than 10 fragments in all samples were removed before differential expression analysis. The differential expression between conditions was statistically assessed by R/Bioconductor package edgeR (version 3.6.2) [21] or DESeq (version 1.16.0) [22]. Genes with a false discovery rate (FDR) ≤0.05 and a length of >200 bp were considered differentially expressed. For gene ontology analysis, IPA (Qiagen) and DAVID version 6.7 [23] were used with gene symbols. Gene set enrichment analysis (GSEA) was carried out by using the curated gene sets (C2) of the Molecular Signature Database version 4.0 provided by the Broad Institute (http://www.broad.mit.edu/gsea) [24]. Note that the list of differentially expressed genes (DEGs) and entire detectable genes derived from each sample were used for IPA and GSEA analysis, respectively. In particular, we followed the standard procedure as described by GSEA user guide (http://www.broadinstitute.org/gsea/doc/GSEAUserGuideFrame.html). FDR <0.25 is statistically significant for GSEA analysis. Detailed information for data sets is given in the supplemental online data.

### Accession Numbers

The RNA‐Seq data (GSE67070) on three pairs of freshly purified human benign prostatic basal and luminal cells was described [11]. The GEO number for RNA‐Seq reported in this paper is GSE74698. All other methods are described in the supplemental online data.

## Results

### Luminal‐Derived Organoid Cells Can Be Transformed to Generate Human PCa

**Figure 1:**
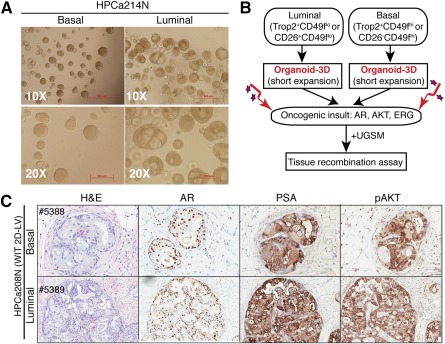
Organoid culture‐enriched luminal progenitor cells can function as a cell of origin for human PCa. **(A):** Phase images of spheres and organoids derived from freshly sorted human benign prostatic basal (Trop2^+^CD49f^hi^) and luminal (Trop2^+^CD49f^lo^) populations cultured in 3D conditions. Note that luminal cells formed typical organoids with hollow lumens whereas basal cells formed solid spheres. Similar results were obtained using CD26‐based basal (CD26^−^CD49f^+^) and luminal (CD26^+^CD49f^−^) cells (data not shown). Scale bars = 500 μm (top) and 200 μm (bottom). **(B):** Schematic of experimental procedures for our transformation assays. **(C):** H&E and immunohistochemistry analysis of indicated cell‐derived human PCa in TR assays. Scale bars, 100 μm (top) and 200 μm (bottom). Abbreviations: 3D, three‐dimensional; H&E, hematoxylin and eosin; PCa, prostate cancer; TR, tissue regeneration; UGSM, urogenital sinus mesenchyme.

Previous studies have established that human prostate basal and luminal epithelial cells can be distinguished by the Trop2^+^CD49f^hi^ versus Trop2^+^CD49f^lo^ phenotype [6, 14] or the CD26^−^CD49f^hi^ versus CD26^+^CD49f^lo^ phenotype [7]. A small population (<2%) of CD26^+^CD49f^lo^ luminal cells has the ability to establish 3D organoids containing both luminal and basal cells, suggesting that these rare organoid‐founding cells may represent bipotential human luminal progenitor cells [7]. Very recently, freshly purified CD26^+^ human prostate luminal cells were shown to be transformed by c‐MYC combined with activated AKT1 in the 3D culture conditions to initiate PCa [12]. We addressed the same question of whether human luminal progenitor cells can function as a cell of origin for PCa by first purifying out Trop2^+^CD49f^lo^ luminal cells and, for comparison, Trop2^+^CD49f^hi^ basal cells, from benign prostate tissue samples and then established 3D organoids (Fig. 1A). We observed that the basal cells formed solid spheres whereas luminal cells developed typical organoids with prominent lumen (Fig. 1A), consistent with others’ results [7]. We then disaggregated the organoids into single cells, infected them with oncogene‐encoding lentiviruses including a GFP‐marked lentivirus carrying AR and an RFP‐marked lentivirus carrying activated AKT and ERG [6], recombined the cells with mUGSM, and then implanted the recombinants in NOD‐SCID‐IL‐2Rγ^−/−^ mice for TR assays [11] (Fig. 1B). As shown in Figure 1C, both luminal and basal cells were transformed by AR/AKT/ERG to generate abnormal structures reminiscent of human primary PCa positive for both AR and PSA but negative for basal cell markers p63 and CK14 (data not shown). Similar results were obtained using other benign sample‐derived basal and luminal cells purified based on CD49 or CD26 phenotypes (Fig. 1B; supplemental online Table 1; data not shown). While this report was in preparation, Park et al. published a study showing similar transformation results with CD26^+^ luminal cells [12]. Taken together, these results demonstrate that luminal prostate progenitors that can establish organoids can function as cells of origin for PCa.

### WIT Medium Readily Propagate Human Prostate Epithelial Cells

Although the development of organoid culture conditions has greatly facilitated the study of normal tissue development in diverse epithelial organs, the system has several caveats. First, the human prostate luminal progenitors are rare (<2%) [7], and generation of a large number of cells derived from luminal progenitors for biological study is still challenging even with organoid system. Second, the organoids represent a mini organ system with presence of both basal and fully differentiated luminal cells [7], thus not suitable for investigation of luminal progenitor cells solely. Therefore, there is still an urgent need for developing a feasible two‐dimensional (2D) culture system that can readily and quickly enrich high numbers of luminal progenitor cells.

**Figure 2:**
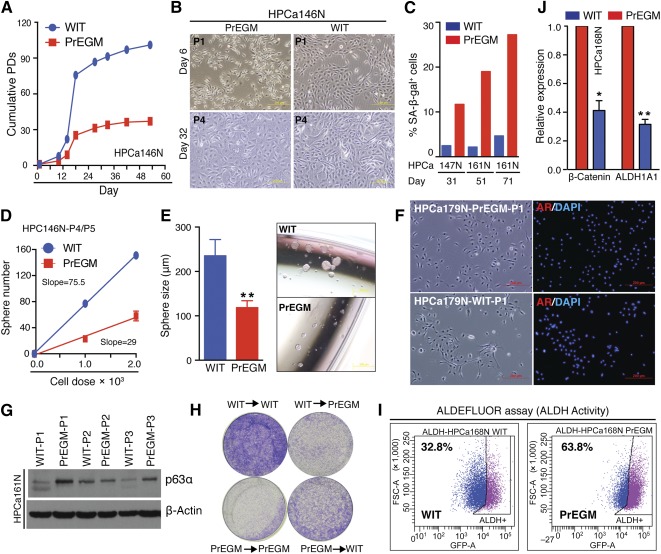
WIT represents a better culture system for human prostate epithelial cells. **(A):** Comparison of cumulative PDs of human prostate bulk epithelial cells cultured in serum‐free PrEGM versus WIT medium. **(B):** Representative phase images showing the morphology of epithelial cell cultures at P1 and P4. **(C):** Quantification of SA‐β‐gal^+^ cells cultured in either WIT or PrEGM at the indicated time points. **(D, E):** Cells cultured in WIT exhibit high stem/progenitor activities in vitro. Shown are the number of spheres formed in a limiting dilution sphere assay **(D)** and quantification of sphere size as well as images of representative spheres **(E)**. **(F):** IF of AR in human benign primary prostate epithelial cells cultured in WIT or PrEGM. **(G)**: Western blot analysis of p63 expression in the indicated populations of human prostate epithelial cells. **(H):** The “medium‐switching” colony formation assay. Equal numbers of cells (4.5 × 10^4^) were plated in either their original or switched medium for a culture of 3–4 days and visualized by 0.1% crystal violet staining. **(I):** ALDEFLUOR assay measuring the stem/progenitor cell frequency in bulk cells cultured in either WIT or PrEGM. **(J):** Quantitative reverse transcription‐polymerase chain reaction analysis of the indicated SC genes in cells cultured in WIT or PrEGM. Image magnification **(B, E, F)** = 10×. Abbreviations: ALDH, aldehyde dehydrogenase; DAPI, 4′,6‐diamidino‐2‐phenylindole; HPCa, human prostate cancer; IF, immunofluorescence; P1, primary culture; P4, passage 4; PD, population doubling; PrEGM, prostate epithelial cell growth medium.

Prostate epithelial cell growth medium (PrEGM) has been widely used to culture basal‐like human prostate epithelial (HPE) cells but it is incapable of propagating luminal cells [25]. Given that the WIT medium is capable of propagating luminal‐like human mammary epithelial cells [13], we reasoned that this medium might be able to expand human prostate luminal progenitor‐like epithelial cells. To test this possibility, we cultured primary HPE cells from disassociated human benign prostate tissues [11] (supplemental online Table 1) in WIT and PrEGM supplemented with 10 μM p160ROCK inhibitor Y‐27632 [26], and then compared their growth kinetics. As observed earlier [25], primary HPE cells cultured in PrEGM gradually lost proliferative capacity and ceased proliferating after approximately 30 population doublings (PDs) (Fig. 2A; supplemental online Fig. 1A). These cells displayed a large and flat morphology, and stained positive for senescence‐associated β‐galactosidase (Fig. 2B, 2C; supplemental online Fig. 1B). In contrast, cells maintained in WIT proliferated faster and reached >100 PDs in approximately 60 days (Fig. 2A; supplemental online Fig. 1A), indicating that WIT extended the HPE cell lifespan albeit cells eventually underwent senescence after prolonged culture for 3–4 months (not shown). These results, together, suggest than WIT is a more robust system than PrEGM for culturing primary HPE cells.

Sphere formation assays, which have been widely used to assess (cancer) stem/progenitor cell activities [7, 8, 9, 11], showed that primary HPE cells grown in WIT possessed higher sphere‐forming efficiency and established larger spheres than cells grown in PrEGM (Fig. 2D, 2E). Immunofluorescence (IF) staining revealed that primary WIT‐cultured bulk HPE cells lacked the AR (Fig. 2F; a positive control for AR staining is shown in supplemental online Fig. 1C) and PSA (not shown) proteins. These two pieces of data, together, suggest that the WIT medium does not propagate fully differentiated AR^+^/PSA^+^ luminal cells but preferentially support cells with stem/progenitor activities.

To determine whether the primary HPE cells cultured in PrEGM and WIT may preferentially enrich different cell (sub)types, we first examined the expression of basal marker p63 and luminal marker CK18 by immunoblotting. We found that cells maintained in WIT had lower p63 and higher CK18 levels than those in PrEGM, especially in the primary (P1) cultures (Fig. 2G; supplemental online Fig. 1D). This implied that WIT might be propagating cells more mature than p63^+/hi^ basal cells. We next performed a “medium‐switching” colony assay and found that the WIT medium always promoted colony formation (Fig. 2H). This suggested that WIT might support the growth of cell subtypes that PrEGM did not. IF analysis showed that the bulk cultured HPE cells in PrEGM and WIT were generally CK5^+^, but more CK8^+^ cells were found in WIT (compare panels 4 and 3) and the difference was further enlarged with passaging (compare panels 6 and 5) (supplemental online Fig. 1E). Considering that PrEGM primarily maintains basal (stem/progenitor) cells [25], we carried out functional ALDEFLUOR assays, a classical approach to measure stem/progenitor cell activities [8]. We found that, as expected, cells cultured in PrEGM contained higher SC content than those cultured in WIT (Fig. 2I). Quantitative reverse transcription‐PCR analysis showed that cells in PrEGM expressed higher levels of SC genes (*β‐catenin* and *ALDH1A1*) compared with those grown in WIT (Fig. 2J). Collectively, these data suggest that both WIT and PrEGM support p63^+^ basal stem/progenitor cells but WIT also supports a population of more mature cells that, likely, are luminal progenitors.

### WIT Captures Human Prostatic Luminal Progenitor Cells That Can Regenerate Prostatic Glands In Vivo

**Figure 3:**
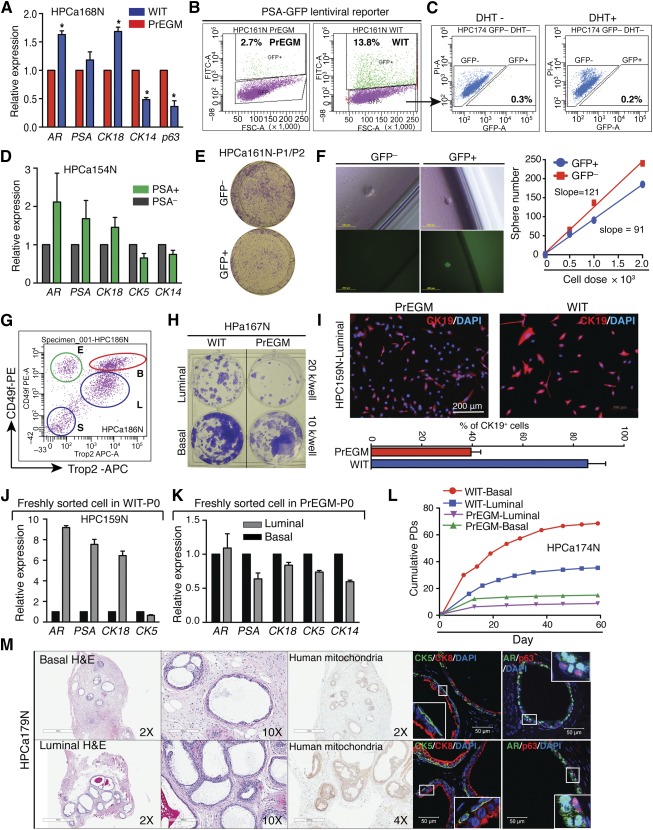
WIT captures luminal progenitor cells that can regenerate prostatic glands in vivo. **(A):** qRT‐PCR analysis of the indicated genes in human benign prostate primary cells cultured in WIT or PrEGM. **(B, C):** FACS analysis of % GFP^+^ cells in PSAP‐GFP lentivirus infected cells originally cultured in either PrEGM or WIT **(B)** and of sorted PSA^−^ cells cultured with or without DHT (10 nM) for an additional 2 weeks. **(D):** qRT‐PCR analysis of indicated genes in purified PSA^−^ and PSA^+^ populations from human benign prostate primary cells cultured in WIT. **(E, F):** PSA^+^ cells exhibit lower stem/progenitor activities in vitro than PSA^−^ cells. Colony formation **(E)** and limiting dilution sphere assays **(F)** are shown. Scale bars = 200 μm **(F)**. **(G):** FACS plots of prostate basal (B), luminal (L), endothelial‐enriched (E), and stromal‐enriched (S) populations identified as Trop2^+^CD49f^hi^, Trop2^+^CD49f^lo^, Trop2^−^cd49f^hi^, and Trop2^−^CD49f^−^, respectively. **(H):** Colony formation assay performed using freshly purified basal and luminal cell populations seeded in the indicated conditions. **(I):** IF analysis of CK19 (upper) and quantification of % CK19^+^ cells (lower) in freshly sorted luminal cells initially expanded in PrEGM or WIT. Scale bars = 200 μm. **(J, K):** qRT‐PCR analysis of indicated genes in primary (without passage) WIT‐ **(J)** and PrEGM‐cultures **(K)** derived from freshly purified human benign prostatic basal and luminal cell populations. **(L):** Comparison of cumulative PDs of freshly purified human prostatic basal and luminal cells cultured in either PrEGM or WIT. **(M):** H&E and human‐specific mitochondria staining, and IF analysis of CK5/CK8 and AR/p63 proteins in prostate tissues regenerated in vivo from primary WIT‐cultures derived from basal and luminal populations purified from HPCa179N. Scale bars = 1 mm (×2 images), 200 μm (×20 images), and 50 μm (confocal images). Abbreviations: DAPI, 4′,6‐diamidino‐2‐phenylindole; DHT, dihydrotestosterone; FACS, fluorescence‐activated cell sorting; H&E, hematoxylin and eosin; IF, immunofluorescence; PD, population doubling; PrEGM, prostate epithelial cell growth medium; qRT‐PCR, quantitative reverse transcription‐polymerase chain reaction.

To test the above suggestion, we analyzed the expression of basal (*CK14*, *p63*) and luminal (*AR*, *PSA*, *CK18*) markers in primary HPE cells cultured in either PrEGM or WIT, and we found that WIT supported a prostatic cell population with increased expression of luminal markers and decreased expression of basal markers (Fig. 3A). To further dissect the cellular heterogeneity of the WIT‐grown cells, we used a PSA promoter (PSAP)‐driven GFP lentiviral reporter system in which the expression of GFP marks relatively mature luminal‐like PSA^+^ cells [9]. Fluorescence‐activated cell sorting (FACS) analysis showed that infected primary HPE cells cultured in PrEGM had a minimal number of GFP^+^ cells, whereas cells cultured in WIT displayed a greater than fivefold increase in GFP^+^ frequency (Fig. 3B; supplemental online Fig. 2A). To rule out the possibility that the difference in percentage GFP^+^ (%GFP^+^) was caused by differences in lentiviral infection efficiency, we simultaneously infected the same number of cells maintained in PrEGM and WIT with a CMV‐dsRed lentivirus [9] at an MOI of 15 overnight. We found that the infection efficiency of cells in PrEGM was actually much higher than in WIT (supplemental online Fig. 2B). To rule out that GFP^−^ basal‐like cells might have contributed to the observed GFP^+^ phenotype through basal to luminal differentiation, we purified out GFP^−^ cells and cultured them in WIT with or without 10 nM dihydrotestosterone (DHT) for an additional 2 weeks. We found that the GFP^−^ cells remained GFP^−^ (Fig. 3C; supplemental online Fig. 2C), indicating the absence of differentiation in these conditions. Indeed, addition of DHT to either culture system did not change the %GFP^+^ (data not shown). These observations suggest that the *PSA* mRNA‐positive (PSA^+^) cells are present in the bulk HPE cultures and can be propagated by WIT. Finally, given the relatively differentiated nature of PSA^+^ cells, we investigated whether the GFP^+^ cells represented a subset of luminal progenitor cells. Quantitative reverse transcription‐PCR indicated that the GFP^+^ population displayed a higher expression of luminal markers than the GFP^−^ population (Fig. 3D). Limiting dilution colony and sphere assays demonstrated that the GFP^+^ cells formed fewer colonies (Fig. 3E) and spheres (Fig. 3F) compared with GFP^−^ cells (supplemental online Fig. 2D), suggesting that the GFP^+^ cells had reduced SC‐like properties. The overall cellular GFP intensity was low (supplemental online Fig. 2C), consistent with the absence of appreciable AR and PSA protein (Fig. 2F) and relatively differentiated nature of luminal progenitors compared with basal/stem cells. Based on these functional properties from the previously discussed SC‐related assays, we define the WIT‐expanded luminal cells as luminal progenitors, and our data, thus far accumulated, establish that, in contrast to PrEGM that mainly supports PSA^−^ basal/stem cells, WIT maintains and propagates not only PSA^−^ but also PSA^+^ luminal progenitor cells.

To strengthen this claim, we FACS‐purified basal/stem (Trop2^+^CD49f^hi^) and luminal (Trop2^+^CD49f^−/lo^) populations [14] and plated them into either WIT or PrEGM. The results showed that both basal and, in particular, luminal cells survived better in WIT (Fig. 3H). We also used CD26 and CD49f to separate epithelial lineages as reported [7, 12] and found that WIT also represented a better system than PrEGM to propagate both CD26^−^CD49f^hi^ basal cells and CD26^+^CD49f^−/lo^ luminal cells (supplemental online Fig. 2E). Furthermore, purified primary luminal cultures in WIT showed a dramatic increase in the frequency and intensity of a luminal progenitor marker CK19 [27, 28, 29] staining (Fig. 3I), and in the expression of luminal genes (*AR*, *PSA*, *CK18*), compared with those in PrEGM (Fig. 3J). When cultured in PrEGM, luminal cells quickly lost their luminal gene expression pattern (Fig. 3K). Analysis of the cell growth kinetics indicated, conclusively, that when cultured in PrEGM, both basal and luminal cells behaved similarly, whereas in WIT, their cell‐type differences were retained (Fig. 3L). The cell growth kinetic analysis also suggested that the WIT medium greatly retained the proliferative potential of both basal/stem and luminal progenitor cells (Fig. 3L), as further supported by the observations that the WIT‐expanded primary HPE cells showed markedly enhanced sphere‐forming ability (supplemental online Fig. 2F). Considering the complex dynamic phenotypic changes associated with primary culture and the cellular plasticity of epithelial stem/progenitor cells in different assays [7, 30], we are currently investigating the identity of the luminal progenitors.

Finally, we confirmed the SC properties of cultured basal cells and luminal progenitors in vivo. By coinjecting HPE cells and mUGSM subcutaneously, we found that both WIT‐cultured primary basal and luminal cells could readily regenerate prostate glandular structures with clearly stratified basal (CK5^+^ and P63^+^) and luminal (CK8^+^ and AR^+^) layers (Fig. 3M). Some regenerated prostatic glands derived from WIT‐expanded luminal cells contained secretions in the lumen (Fig. 3M). The human origin of the structures was verified by human‐specific mitochondria staining. Similar results were observed in other basal and luminal HPE cells (supplemental online Fig. 2G).

### Distinct Transcriptomes of Naïve Basal and Luminal Cells, and Culture‐Enriched Lineage‐Specific Stem/Progenitor Cells

The establishment of WIT (compared with PrEGM) as a robust culture system for HPE stem/progenitor cells, especially for luminal progenitor‐like cells, provided us the unique opportunity to interrogate the genome‐wide gene expression profiles in these cell types. To this end, we examined the transcriptomes of bulk HPE (supplemental online Fig. 3A) and purified basal and luminal (supplemental online Fig. 3B) cells short‐term cultured in PrEGM or WIT for 7–9 days. By sequencing ribosomal RNA depleted RNAs, we obtained an average of 196.1 million reads per sample with an average mapping rate of 94% to the reference human genome (hg38) (supplemental online Fig. 3C), indicating high sequencing depth and quality (supplemental online Fig. 3D). We have recently performed whole‐genome profiling of unperturbed human benign prostatic basal and luminal cells (hereafter referred to as naïve basal and luminal cells) [11]. We compared the RNA‐Seq data of culture‐enriched lineage stem/progenitor cells to that of naïve basal and luminal cells. Unsupervised hierarchical clustering (supplemental online Fig. 3E) showed that (a) freshly purified naïve cells clustered separately from cultured cells; (b) cells cultured in the same medium clustered together, supporting the idea that WIT and PrEGM are distinct systems each propagating different HPE cell types; (c) bulk and basal cultures were grouped more closely together than either was with luminal cultures, consistent with the fact that the basal layer contains the main pool of prostate SCs [5, 30]; and (d) luminal cultures were separated from bulk and basal cultures in both systems with a greater difference captured by WIT, confirming that WIT is better suited for culturing luminal progenitor cells.

**Figure 4:**
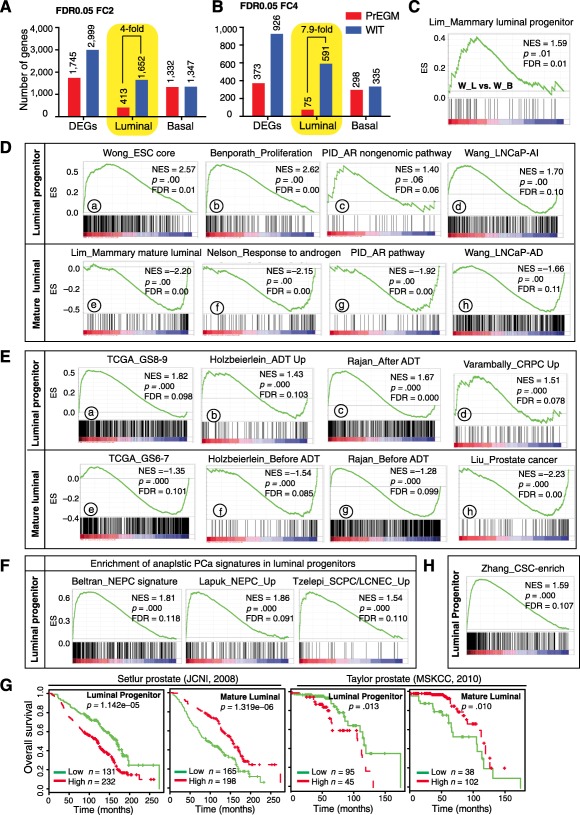
WIT‐expanded luminal progenitor cells have a distinct transcriptomic profile resembling that of aggressive prostate cancer. **(A, B):** Transcriptomic evidence that WIT enriches luminal cell‐specific genes. Shown are the DEGs extracted from indicated culture systems when comparing luminal to basal cell cultures at a statistical threshold of FDR <0.05 and FC ≥2 **(A)** or FC ≥4 **(B)**. **(C):** GSEA showing similarities in gene signature in WIT‐cultured prostatic luminal cells and mammary luminal progenitors. W_L and W_B, primary WIT‐cultures of freshly purified human prostatic luminal and basal populations, respectively. **(D–F):** GSEA showing enrichment of the indicated gene signatures in luminal progenitor cells (W_L) compared with freshly purified luminal (Trop2^+^CD49f^lo^) cells (mature luminal). Note that an FDR <0.25 is statistically significant for GSEA analysis. **(G):** Meta‐analysis showing higher level of luminal progenitor signature and lower level of mature luminal signature correlating with reduced overall patient survival, respectively. Data were based on the Setlur and Taylor studies. **(H):** GSEA showing the enrichment of human prostate cancer stem cell‐enriched gene signatures in luminal progenitor cells compared with freshly purified luminal cells. Abbreviations: DEGs, differentially expressed genes; ES, enrichment score; FDR, false discovery rate; GSEA, gene set enrichment analysis; NES, normalized enrichment score.

To “zoom in” on the molecular differences between luminal and basal cultures grown in the same medium, we focused on the DEGs. As shown in Figure 4A, by applying a stringent statistic threshold of <0.05 and a fold change (FC) ≥2, we found that the molecular difference between basal and luminal cultures was greater when cultured in WIT compared with PrEGM (supplemental online Table 2), as reflected by the total number of DEGs. This difference was mainly maintained by luminal DEGs, as a similar number of basal cell DEGs was observed in both systems (Fig. 4A). Because of the large number of DEGs when using FC ≥2, we increased the threshold to FC ≥4, and found a similar pattern except that the difference in luminal cell DEGs was further widened (from fourfold to eightfold; Fig. 4B). Furthermore, GSEA indicated that, when luminal cells and basal cells cultured in WIT were compared with known gene expression signatures, the luminal cells took on the gene signature of mammary luminal progenitors (Fig. 4C). These results, collectively, indicate that culture‐enriched prostatic basal SC and luminal progenitors express dramatically different gene expression profiles and that WIT represents a better system to propagate luminal progenitors.

We investigated the transcriptomic differences between WIT‐enriched luminal progenitors versus mature luminal cells [11] (Fig. 4D) and found that luminal progenitors were enriched in signatures associated with SC and cell proliferation (Fig. 4Da, Fig. 4Db, respectively) whereas mature luminal cells were enriched in signatures associated with mature mammary luminal cells (Fig. 4De), androgen responsiveness (Fig. 4Df), active AR pathway (Fig. 4Dg), and androgen‐dependent PCa cells (LNCaP‐AD) (Fig. 4Dh). An AR nongenomic pathway signature was enriched in luminal progenitors (Fig. 4Dc), consistent with the fact that these cells were androgen‐independent but possessed low AR activity evidenced by partially PSA^+^ (Fig. 3B, 3D). The enrichment of the LNCaP‐AI (androgen independent) signature (Fig. 4Dd) further confirmed the progenitor nature of luminal cultures in WIT, as we have previously shown that LNCaP‐AI greatly enriches PSA^−^ cells that have SC properties [9].

### Luminal Progenitor Gene Expression Profile Is Linked to PCa Aggressiveness, Castration Resistance, Metastatic Propensity, and Poor Patient Survival

The majority of untreated primary PCa present as adenocarcinomas with a luminal‐like phenotype, whereas a small subset (1%–5%) is classified as undifferentiated or anaplastic PCa variants known as small cell PCa (SCPC) or neuroendocrine PCa (NEPC), which are generally AR‐negative, have a clinically aggressive behavior and are significantly increased (up to 25%) during castration‐resistant PCa (CRPC) progression [31]. GSEA showed that typical adenocarcinoma (Fig. 4Eh) and treatment naïve (Fig. 4Eg, 4Ef; supplemental online Figure 3Fc) PCa presented a mature luminal cell gene expression profile. We extracted two gene signatures corresponding to patients with low and high Gleason Score (GS) from the TCGA PCa data [11] and found that although the low GS signature was enriched in mature luminal cells (Fig. 4Ee), the high GS signature was significantly enriched in luminal progenitor cells (Fig. 4Ea). Furthermore, the luminal progenitor cell profile was greatly enriched in gene signatures associated with resistance to androgen‐deprivation therapy (ADT; Fig. 4Eb, 4Ec; supplemental online Figure 3Fa) and CRPC (Fig. 4Ed; supplemental online Figure 3Fb), suggesting a global luminal progenitor‐like molecular feature for these aggressive PCa. A 19‐gene indolent PCa signature [32] was enriched only in mature luminal cells (supplemental online Figure 3Fd), suggesting a predictive value of the luminal progenitor gene profile to distinguish indolent versus aggressive disease. Remarkably, anaplastic PCa signatures derived from currently available datasets were all dramatically enriched in the luminal progenitor cells (Fig. 4F). Collectively, these results link luminal progenitor gene profile to aggressive and castration‐resistant PCa and establish luminal progenitor‐related gene signature as a potential indicator of lethal PCa.

Next, we stratified PCa patients based on similarities in gene expression to luminal progenitor cells or mature luminal cells and compared their clinical outcomes. To facilitate this analysis, based on the overlapping of DEGs (supplemental online Table 3) and the multiple GSEA leading edges shown in Figure 4E and 4F, we created two gene signatures (supplemental online Table 4) corresponding to luminal progenitors (266 genes) and mature luminal cells (203 genes), respectively. Two large datasets [33, 34] were interrogated to show that patients whose cancer gene expression most closely matched the luminal progenitors or mature luminal cells had a worse or a better survival, respectively (Fig. 4G). Finally, Oncomine concept analysis showed that 124 of the 266 genes in luminal progenitor signature and 71 of the 203 genes in mature luminal signature were upregulated and downregulated in metastatic versus primary PCa (supplemental online Fig. 3G), suggesting that metastatic PCa are more likely to express a luminal progenitor‐like profile. Furthermore, 55 genes in the luminal progenitor signature and 31 in the mature luminal signature were positively and negatively correlated with PCa recurrence, respectively (supplemental online Fig. 3H), further supporting luminal progenitor gene signature as an adverse predictor of patient survival.

Finally, we performed RNA‐Seq analysis on matched cancer stem cell (CSC)‐enriched and non‐CSC populations directly isolated from five high‐involvement tumor specimens. It has recently been shown that CD49f is an efficient marker for stem‐like cancer cells in human PCa [35], and a small population of CD49f^+^ cells exists in almost all clinical PCa samples examined by immunohistochemistry (IHC) analysis [36]. Therefore, we employed CD49f, together with Trop2 as a pan‐epithelial marker, as a CSC enrichment marker to separate tumor cells into matched CSC‐enriched population (CD49f^hi^Trop2^+^) and non‐CSC population (CD49f^−/lo^Trop2^+^). Two signatures corresponding to each population were generated (supplemental online data). GSEA indicated that the CSC signature was significantly enriched in luminal progenitor cells whereas the non‐CSC signature predominated in mature luminal cells (Fig. 4H, data not shown). Altogether, these analyses indicate that the luminal progenitor gene expression profile is linked to aggressive PCa subtypes, adverse patient outcomes and poor patient survival.

### Culture‐Enriched Luminal Progenitors Can Function as a Cell of Origin for PCa

**Figure 5:**
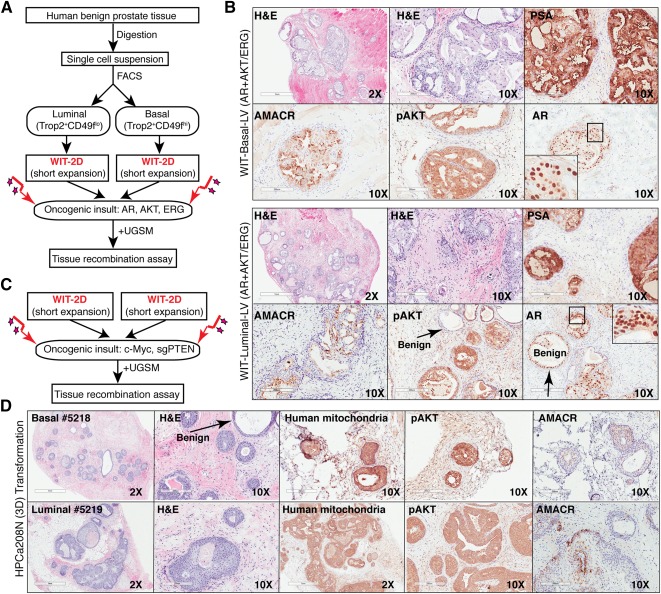
Luminal progenitors as cells of origin for PCa. **(A, C):** Schematic of experimental procedures. **(B, D):** H&E and immunohistochemistry analysis of indicated cell‐derived human PCa in tissue regeneration assays. For B, the top panels (WIT‐Basal) were results from HPCa208N and the bottom panels (WIT‐Luminal) from HPCa207N. Scale bars = 1 mm (×2 images) and 200 μm (×10 images). Abbreviations: 2D, two‐dimensional; 3D, three‐dimensional; FACS, fluorescence‐activated cell sorting; H&E, hematoxylin and eosin; PCa, prostate cancer; UGSM, urogenital sinus mesenchyme; sgPTEN, single guide PTEN.

Finally, we evaluated whether WIT‐expanded luminal progenitor cells can serve as a cell of origin for PCa. We transduced the WIT‐enriched luminal progenitors with the same (Fig. 1B) oncogene‐encoding lentiviruses encoding AR and AKT/ERG (Fig. 5A), followed by subcutaneous injection of the infected primary cells mixed with mUGSM cells in Matrigel into NOD‐SCID‐IL‐2Rγ^−/−^ mice. Primary basal cell cultures were used as the “positive” control. Both WIT‐propagated basal/stem and luminal progenitor populations could be transformed, with WIT‐luminal cells showing lower efficiency (13% vs. 9.4%; *n* = 192 injections), to form abnormal prostatic structures resembling human primary PCa with high levels of AR and PSA expression (Fig. 5B). No basal cells were observed in tumor areas (data not shown). The GFP/RFP signals and human‐specific mitochondria staining verified the human origin of the prostatic tumors (supplemental online Fig. 4A). Similar results were obtained using other benign tissue‐derived basal and luminal cultures (supplemental online Fig. 4B).

To induce oncogenesis by other means, we combined a lentivirus expressing c‐MYC [15] and another lentivirus coexpressing a mammalian codon‐optimized Cas9 nuclease along with a single guide RNA (sgRNA) targeting *PTEN* [16] (Fig. 5C). The sequence of the sgRNA (supplemental online Fig. 4C) was previously described [37]. After 2–3 months, we observed, from both WIT‐cultured primary basal/stem cells and luminal progenitors, the development of tumor‐like structures that stained positive for pAKT and AMACR, indicating the loss of PTEN in cancerous tissues (Fig. 5D). Cells transformed by both AR/AKT/ERG and c‐MYC/sgPTEN generated heterogeneous tumors including adenocarcinoma and adenosquamous carcinoma (Fig. 5B, 5D), consistent with a recent report [38].

## Discussion

**Figure 6:**
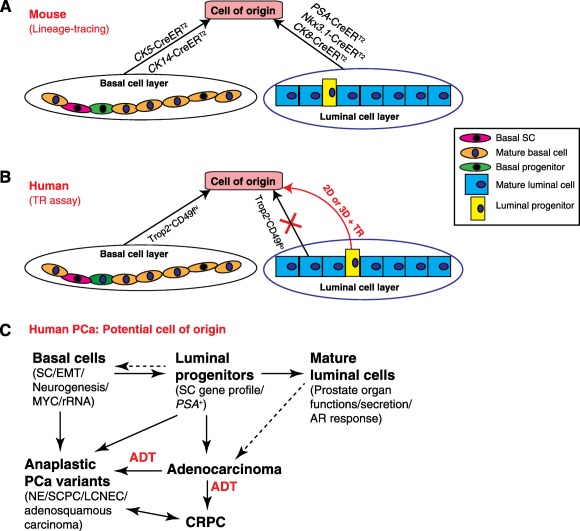
Luminal progenitors as a potential cell of origin for human PCa. **(A):** Schematic depicting both (bulk) basal and luminal cells can function as the targets of tumorigenic transformation based on lineage tracing studies in the mouse prostate. It is unclear whether the putative luminal progenitor cell (yellow; right) can be transformed. **(B):** In the TR assays using purified human (bulk) prostatic basal and luminal cells, only basal cells can be transformed to form tumors. We show, from the present study, that the luminal progenitor cells enriched by WIT culture can serve as a cell of origin for tumor development (red arrow). **(C):** Schematic depicting potential involvement of different cell types in generating different subtypes of human PCa. It is not known whether our luminal progenitors can regenerate basal/stem cells and whether mature luminal cells can give rise to PCa. Further information is given in the Discussion. Abbreviations: 2D, two‐dimensional; 3D, three‐dimensional; ADT, androgen‐deprivation therapy; CRPC, castration‐resistant prostate cancer; LCNEC, large‐cell neuroendocrine carcinoma; NE, neuroendocrine; PCa, prostate cancer; SC, stem cell; TR, tissue regeneration; PCa, prostate cancer; SCPC, small cell PCa.

Lineage tracing studies in mice, using *CK5*‐ or *CK14*‐Cre to mark basal cells or using *PSA‐*, *CK8‐*, or *Nkx3.1*‐Cre to mark luminal cells have demonstrated that both basal and luminal cells can be tumorigenically transformed (e.g., by *PTEN* loss) to form tumors (Fig. 6A) [3, 39, 40]. However, one early TR‐based study [6] using freshly purified bulk human prostatic basal and luminal cells shows that only the Trop2^+^CD49f^hi^ basal cells can serve as the targets of tumorigenic transformation (Fig. 6B, left). One possibility is that the stem/progenitors cells in the luminal cell layer of the human prostate competent for tumorigenic transformation are very rare. Indeed, the CD26^+^CD49f^lo^ human prostate luminal population harbors only <2% of the bipotential progenitor cells that can establish 3D organoids that contain both basal and luminal cells [7]. Organoids derived from freshly purified CD26^+^ human prostate luminal cells first infected with c‐MYC/AKT1 can be tumorigenically transformed to initiate PCa [12]. Our current study also reveals that the Trop2^+^CD49f^lo^ luminal cell‐derived organoids can be tumorigenically transformed by AR/AKT/ERG. These two complementary studies [12; this study] provide strong evidence that the small population of human prostate luminal cells that can establish 3D organoids (i.e., luminal progenitors, can also function as the cell‐of‐origin for PCa [Fig. 6B; right]).

The major significance of our current study is that we have reported a simple 2D culture system that can greatly expand primary human prostate epithelial progenitor cells. By adopting and modifying the WIT medium that has previously been developed to culture human mammary luminal progenitors [13], we show that WIT, unlike PrEGM and some other culture systems (e.g., fibroblast feeder‐based culture [41] and low Ca^2+^ serum‐free defined medium [42]), can efficiently maintain and propagate the human prostatic luminal progenitors that possess enormous proliferative potential and sphere‐forming capacities, express luminal genes (*AR, PSA*, and *CK18*) and have AR activity evidenced by *PSAP‐*GFP reporter assay. When put into 2D culture, luminal progenitors grow better in WIT compared with the 3D organoid medium (data not shown). Using the short‐term WIT‐expanded cultures, we demonstrate that luminal progenitor cells in the freshly purified Trop2^+^CD49f^lo^ or CD49f^lo^CD26^+^ cell population can be tumorigenically transformed by 2 genetic contexts (i.e., AR/AKT/ERG and c‐MYC/sgPTEN [Fig. 6B, right]).

We termed the WIT‐propagated luminal cells as “luminal progenitors” mainly based on their functional properties displayed in various experimental assays. For example, freshly purified luminal cells briefly propagated in WIT, when transplanted with mUGSM under the kidney capsule, could regenerate prostatic glands that contain both differentiated AR^+^ luminal cells and CK5^+^ basal cells. These short‐term WIT expanded cells are highly proliferative, and overexpress luminal differentiation markers at the mRNA levels. This definition is analogous to others’ [7] in which the authors termed a small population of (phenotypically undefined) luminal cells that grew up in a 3D culture system as multipotent progenitors because they functionally exhibited SC properties. Other functional assays also indicate that our 2D WIT‐expanded cultures are enriched in luminal progenitors. Currently, there is no well‐accepted definition for prostate luminal progenitors, and no studies have reported the phenotypic features (e.g., cell surface markers) of luminal progenitors in either human or mouse prostate [7, 40, 43]. Although lineage‐tracing studies implicate a cell population(s) in the luminal layer capable of self‐duplicating, the identity of these cells is unclear. A luminal progenitor population has been defined as Lin^−^CD24^hi^CD29^lo^CD61^+^CD133^−^ in the mouse mammary gland [44]; however, it is unclear whether this marker profile is applicable to the human prostate luminal cells.

The ability of the enriched luminal progenitor cells in either 3D or 2D culture to initiate PCa (Fig. 6B, right) is unlikely caused by the potential contributions from the “contaminated” basal cells, which represent the SC pool of the prostate tissue [11], because WIT‐expanded luminal and basal cells still display distinct molecular and biological features. Our findings are consistent with the recent evidence for human prostatic luminal progenitor cells that can initiate organoids [7] and with the lineage‐tracing studies [4, 5, 10] showing that both murine prostatic basal and luminal cell layers contain lineage‐restricted stem/progenitor cells that can initiate PCa in different genetic contexts.

Of clinical significance, through comparative analysis of RNA‐Seq data derived from human mature prostatic luminal cells [11] and WIT‐enriched luminal progenitor cells, we link the luminal progenitor gene expression profile to advanced, aggressive, and castration‐resistant PCa subtypes (Fig. 6C). By surveying almost all “eligible” datasets in Oncomine and the current TCGA PCa project, we find that many genes upregulated in luminal progenitors are also commonly overexpressed in aggressive PCa variants (Fig. 4; supplemental online Fig. 3). This molecular resemblance is of great clinical importance as it provides a common molecular understanding for these diverse and poorly characterized aggressive PCa subtypes. Furthermore, we have derived a luminal progenitor gene signature that is predictive of adverse prognosis and poor patient survival. These observations, together, reinforce the biological importance and clinical relevance of our study in that pathways involved in luminal progenitor cell function and self‐renewal may likely play a role in tumor cell survival and disease recurrence after failing ADT.

## Conclusion

Together with others’ recent work [12], our current study fills an important gap in our knowledge regarding the potential cell of origin for human PCa (Fig. 6C). Basal/stem cells, which preferentially express genes associated with SC, EMT, neurogenesis and MYC‐regulated rRNA biogenesis, may represent the cell of origin for anaplastic PCa variants encompassing many morphological subtypes such as NE/SCPC/LCNEC [11] (Fig. 6C). Luminal progenitors, also preferentially expressing SC genes, may give rise to common adenocarcinomas (based on functional transformation assays) as well as anaplastic PCa (based on molecular resemblance) (Fig. 6C). This latter scenario is analogous to BRCA1 basal‐like breast cancers that actually originate from the luminal progenitors and exhibit an overall luminal‐progenitor‐like gene expression profile [45]. Both basal/stem and luminal progenitor cell gene expression profiles are greatly enriched in CRPC, a significant fraction of which manifest clinical features of anaplastic PCa [31] (Fig. 6C). Therefore, our studies ([11]; this study) indicate that the gene expression profiles of basal/stem cells and luminal progenitors likely represent a molecular feature commonly shared by advanced, aggressive, and castration‐resistant PCa that bode poor patient survival. Finally, we envision that the simple 2D culture system reported herein that can rapidly expand NHP progenitor‐like cells should greatly facilitate biological and biochemical studies as well as high‐throughput screening in these cells and in progenitor‐like PCa cells.

## Author Contributions

D.Z.: conception and design, collection and/or assembly of data, data analysis and interpretation, manuscript writing, final approval of manuscript; K.L., Y.L., Y.Z., H.‐P.C., and J.S.: data analysis and interpretation, final approval of manuscript; K.R. and T.C.‐D.: provision of study material or patients, final approval of manuscript; D.G.T.: conception and design, financial support, data analysis and interpretation, manuscript writing, final approval of manuscript.

## Disclosure of Potential Conflicts of Interest

The authors indicated no potential conflicts of interest.

## Supporting information

Supporting Information

Supporting Information

Supporting Information

Supporting Information
