# Supplementary material for: Developing a Novel Two‐Dimensional Culture System to Enrich Human Prostate Luminal Progenitors that Can Function as a Cell of Origin for Prostate Cancer
Source: Stem Cells Transl Med. 2016 Sep 29;6(3):748–60. doi: 10.5966/sctm.2016-0243 (PMC5442765; doi:10.5966/sctm.2016-0243)
Supplement: Supplementary file 1 — Supporting Information [file SCT3-6-0748-s001.pdf]

Developing a novel 2D culture system to enrich human prostate luminal progenitors that can function as a cell of origin for prostate cancer

Dingxiao Zhang, Kevin Lin, Yue Lu, Kiera Rycaj, Yi Zhong, Hsueh-Ping Chao, Tammy Calhoun-Davis, Jianjun Shen, and Dean G. Tang

Inventory of Supporting Information

Supplementary Materials and Methods

Supplementary References

Supplementary Figures

Supplementary Fig. 1, related to Fig. 2

Supplementary Fig. 2, related to Fig. 3

Supplementary Fig. 3, related to Fig. 4

Supplementary Fig. 4, related to Fig. 5

Supplementary Table 1, Primary benign human prostate tissue samples used in the current study

Supplementary Table 2, DEGs in basal and luminal epithelial cells cultured in WIT and PrEGM (4 tabs)

Supplementary Table 3, DEGs in freshly purified and WIT cultured prostatic luminal cells

Supplementary Table 4, Human prostate luminal progenitor and mature luminal signatures

Supplementary Materials and Methods

Human primary prostate tissue processing, FACS, and culture

All primary benign prostate tissues used in the present study were obtained from human PCa (HPCa) patients (Supplementary Table 1) undergoing Da Vinci based radical prostatectomy with the written

informed consent in accordance with federal and institutional guidelines and with the approved IRB protocol (MDACC LAB04-0498). HPCa processing protocol was described previously [1]. The final dissociated single cell suspension was stained with PE-CD49f, FITC-CD26, APC-Cy7-CD45 (eBioscience) and APC-Trop2 antibodies (R&D Systems). FACS analysis and sorting were performed using BD FACSAria™ Fusion (BD Biosciences). Propidium iodide was added prior to FACS analysis to separate viable from dead cells. The $CD45^{+}$ immune cells were excluded, and $Trop2^{+}$ epithelial cells were separated according to high ($Trop2^{+}CD49f^{hi}$, basal-enriched) or low ($Trop2^{+}CD49f^{lo}$, luminal-enriched) expression of CD49f. Alternatively, we also purified the basal ($CD49f^{+}CD26^{-}$) and luminal ($CD49f^{-}CD26^{+}$) fractions using the combination of CD26 and CD49f [2]. To culture either the bulk or purified prostate epithelial cells, cells were plated in 6-well plates or T25 flasks precoated with PureCol (Advanced BioMatrix). PrEGM (Prostate Epithelial Cell Growth Medium) (Lonza) and WIT-P (Stemgent, Cat # 00-0045-500) media were used in this study, both of which were supplemented with 10 µM of p160ROCK inhibitor Y-27632 dihydrochloride (Selleckchem) to inhibit anoikis. For cell passaging, the Trypsin-EDTA for Primary Cells (ATCC, PCS-999-003) and Trypsin Neutralizing Solution (ATCC, PCS-999-004) were utilized. To enrich the stem/progenitor populations from both lineages, the 3D organoid culture system was adopted as described [2].

Colony and sphere formation assays

For colony formation assays [3], we plated primary prostatic cells at a low density (i.e., 800-1000 cells/well) in a precoated 6-well dish, and let cells grow for 7-9 days before the visualization of the culture by crystal violet staining. For sphere-formation assays [4], cells were suspended in 1:1 Matrigel (BD Biosciences)/media in a total volume of 100 µl. The mixtures were then plated around the rims of wells in a 12-well plate and allowed to solidify in 37°C incubator for 25 minutes, followed by addition of 1 ml of warm WIT or PrEGM medium. Usually 7-9 days after plating, spheres with a diameter over 50 µm were counted. For all above experiments, we ran a minimum of triplicate wells for each condition and repeated experiments in different patient-derived cells whenever feasible.

ALDEFLUOR assay

The ALDEFLUOR kit (Stem Cell Technologies) was used to identify the population with high ALDH enzymatic activity using a BD LSRFortessa™ cell analyzer (BD Biosciences), as previously described [5]. Briefly, cells

were incubated at 37°C in ALDEFLUOR assay buffer containing ALDH substrate (1 μmol/L per 1X10^6 cells). As negative control for each experiment, we added 50 nmol/l diethylaminobenzaldehyde (DEAB, a specific ALDH inhibitor) to the cell suspension. The sorting gates were established using propidium iodide (PI)–stained cells for viability.

Histology, immunofluorescence (IF) staining, and microscopy

H&E and IF staining was performed on either 5-μm paraffin-embedded or OCT frozen sections. Basic IF procedures have been described previously [3, 6]. For staining of cell cultures, cells were first grown on glass coverslips precoated with PureCol (Type I collagen) (Advanced BioMatrix), then fixed with 4% paraformaldehyde (PFA) containing 5% sucrose (pH 7.2). The coverslips or the tissue slides were blocked with Background Sniper (Biocare Medical) for 30 minutes, followed by primary antibody incubation overnight at 4°C. Primary antibodies used in this study included: AR (441, Santa Cruz), p63 (4892, Cell signaling), β-actin (A1978, Sigma), CK5 (PRB-1609, Covance), CK8 (TROMA-I, Developmental Studies Hybridoma Bank), CK19 (TROMA-III, Developmental Studies Hybridoma Bank), PSA (A0562, Dako), Phospho (Ser 473)-AKT (sc-7985-R, Santa Cruz), human-specific mitochondria (MAB1273, Chemicon), and AMACR (M3616, Dako). Slides were then incubated with secondary antibodies (diluted 1:700 in antibody diluent (Dako, Carpinteria, CA)) labeled with AlexaFluor 488 or 594 (Invitrogen/Molecular Probes, Grand Island, NY). After washing (3X) with PBS, sections were counterstained with 4,6-diamidino-2-phenylindole (DAPI) (Sigma) and mounted with ProLong® Gold Antifade Mountant (Life Technologies). IHC and IF images were captured by Olympus IX71 and Zeiss LSM510 META confocal microscope, respectively.

Western blotting, SA-β-gal staining and tissue regeneration assays

For Western blotting and SA-β-gal staining, basic procedures have been described [7, 8]. For tissue regeneration assays [9], briefly, varying number of human cells was combined with mouse UGSM in Matrigel or collagen and injected either under the skin or kidney capsule. The outgrowths were harvested for analysis from the experimental mice 3 months later.

RNA isolation and quantitative RT-PCR (qPCR)

Total RNA was isolated from cells using the RNeasy mini kit (Qiagen). The first-strand cDNA synthesis was

achieved by reverse transcription of RNA using random hexamers and SuperScript III Reverse Transcriptase (Invitrogen). Quantitative RT-PCR was performed using the iQ™ SYBR® Green supermix (BioRed) on a 7900HT Fast Real-Time PCR System (ABI, Applied Biosystems). Generally, the housekeeping gene *GAPDH* or *β-actin* was used as internal control for gene expression normalization. The following primers were used for qPCR: *GAPDH* (forward, ACTTTGGTATCGTGGAAGGACT; reverse, GCCTTGGCAGCGCCAGTAG), *β-actin* (forward, CGTGGACATCCGCAAAGAC; reverse, GGAAGGTGGACAGCGAGGC), *β-Catenin* (forward, CCACCCTGGTGCTGACTATC; reverse, ATTTACAGGTCAGTATCAAACCAG), *ALDH1A1* (forward, GCTCTGAGTTTGTTCATCCAATCG; reverse, TGAGTAGGACAGGTAAGTCTGGCG), *AR* (forward, GAGAAGCCTTAGAATGGGTGG; reverse, TGGCTTATGGGATAGGACAAC), *PSA* (forward, GGGAGGGTCTTCCTTTGGCA; reverse, ATCTGAGGGTTGTCTGGAGGA), *CK18* (forward, AACAGCCTGAGGGAGGTGGA; reverse, CTGTCCAAGGCATCACCAAG), *CK14* (forward, AGGAGATCGCCACCTACCGC; reverse, CTGGGCAGCCTCAGTTCTTG), *CK5* (forward, CTGGTCCAACTCCTTCTCCA; reverse, GGAGCTCATGAACACCAAGC), and *p63* (forward, CCTTACTTTGCTGAGGGTTTGA; reverse, TTACCCTGGCTACTCATACACTC).

Generation of gene expression signatures from published literatures and TCGA-PCa RNA-Seq data

The majority of the gene signatures used in this study were obtained from MSigDB, unless noted in the main text or herein. For datasets or signatures from published literatures, we collected them from corresponding supplementary information as described previously [10]. ***FIRST***, through comparative RNA-seq analysis of androgen-dependent prostate cancer cells (LNCaP-AD) versus androgen-independent cancer cells (LNCaP-AI), Wang and colleagues identified gene signatures corresponding to LNCaP-AD and –AI conditions (Fig. 4Dd and 4Dh), respectively [11]. ***SECOND***, by performing the expression profiling study, Liu and colleagues obtained a signature (Fig. 4Eh) over-represented in prostate tumor samples compared to benign tissues [12]. ***THIRD***, Androgen-deprivation therapy (ADT) is the current standard treatment for locally advanced or metastatic PCa. To gain insight into the biology of the androgen responses and the mechanisms of therapy resistance, Holzbeierlein and colleagues performed a microarray analysis and identified gene expression changes that occurred during androgen ablation therapy (goserelin and flutamide) and in resistant disease (Fig. 4Eb and 4Ef, respectively) [13]. ***FOURTH***, Rajan and colleagues recently performed RNA-Seq analysis on tumor-rich, targeted prostatic biopsies from 7 patients with locally

advanced or metastatic PCa before and approximately 22 weeks after ADT initiation [14]. Through the comparison of gene expression profiles, they showed that ADT induced a major shift in gene expression and two gene signatures corresponding to before (Fig. 4Ec) and after (Fig. 4Eg) treatment were reported. ***FIFTH***, Lehmusvaara and colleagues studied the molecular mechanisms of hormonal therapy by comparing the effect of bicalutamide (anti-androgen) and no therapy, followed by radical prostatectomy. They found differentially expressed genes in PCa before and after treatment (Supporting Information Fig. 3Fa and 3Fc) [15]. ***SIXTH***, to characterize the molecular features of clinical hormone-refractory PCa (HRPC or CRPC), Tamura and colleagues analyzed gene expression profiles of 25 clinical HRPCs and 10 hormone sensitive PCa by genome-wide cDNA microarrays and identified 36 up-regulated genes in HRPC (Supporting Information Fig. 3Fb) [16]. ***SEVENTH***, Varambally and colleagues utilized an integrative genomic and proteomic analysis to identify a signature (Fig. 4Ed) of metastatic progression [17]. ***EIGHTH***, by using a bioinformatics approach, Irshad and colleagues established a 19-gene signature (Supporting Information Fig. 3Fd) predictive of indolent PCa with beneficial prognosis [18]. To date, there is only a limited number of studies focused on anaplastic PCa including neuroendocrine PCa (NEPC), small cell prostate carcinoma (SCPC), and large-cell neuroendocrine carcinoma (LCNEC). We were able to retrieve 3 studies with available and relatively large datasets useful to generate anaplastic PCa gene signatures (Fig. 4F). ***NINETH***, Beltran and colleagues profiled 7 NEPC and 30 PCa (i.e., prostate adenocarcinoma), and defined signatures specific for each type [19]. ***TENTH***, Tzelepi and colleagues compared the expression profiles of SCPC and large-cell neuroendocrine carcinoma (LCNEC) xenografts to those of typical prostate adenocarcinoma xenografts, and identified a SCPC/LCNEC signature [20]. ***ELEVENTH***, Lapuk and colleagues reported a molecular signature of castration-resistant NEPC [21]. ***FINALLY***, we recently described the procedure of extracting clinically relevant signatures from the TCGA PCa data [10]. Briefly, the TCGA PCa (PRAD) project currently contains 498 cases in total, of which 487 cases were useful in our analysis according to the availability of the matched RNA-Seq and clinical data. To create gene signatures specific to low (n=605 genes) (Fig. 4Ea) and high (n=639 genes) (Fig. 4Ee) GS patients, we used all detectable genes (n=20,502) and objectively applied them to the shrunken centroid supervised algorithm (PAMR) to perform feature selection [22], resulting in 1244 genes that were associated with GS based on the prediction error.

Generation of prostate CSC and non-CSC Signatures from Primary Human PCa Samples

CD49f is an efficient marker for stem-like cancer cells (or cancer stem cells, CSCs) in human PCa [23], and a small population of $CD49f^{+}$ cells exists in almost all clinical PCa samples examined [24]. Here we employed CD49f as a CSC enrichment marker, coupled with Trop2 (a pan-epithelial marker), to separate tumor cells. We purified matched CSC-enriched population ($CD49f^{hi}Trop2^{+}$) and non-CSC-enriched population ($CD49f^{-/lo}Trop2^{+}$) from 5 PCa samples with ~100% tumor involvement and performed RNA-Seq in the 2 populations (Zhang et al., manuscript in preparation). Based on the DEGs of these two populations, we extracted 2 molecular signatures corresponding to CSC and non-CSC cells (Fig. 4H; data not shown).

Statistical Analysis

Graphpad Prism software was used to calculate mean and standard deviation. In general, the student's *t*-test was used to calculate the statistical significance between the two groups of data. $P < 0.05$ is considered statistically significant.

Supplementary References

1 Goldstein AS, Drake JM, Burnes DL et al. Purification and direct transformation of epithelial progenitor cells from primary human prostate. Nat Protoc. 2011;6:656-667.

2 Karthaus WR, Iaquinta PJ, Drost J et al. Identification of multipotent luminal progenitor cells in human prostate organoid cultures. Cell. 2014;159:163-175.

3 Zhang D, Jiang P, Xu Q et al. Arginine and glutamate-rich 1 (ARGLU1) interacts with mediator subunit 1 (MED1) and is required for estrogen receptor-mediated gene transcription and breast cancer cell growth. J Biol Chem. 2011;286:17746-17754.

4 Xin L, Lukacs RU, Lawson DA et al. Self-renewal and multilineage differentiation in vitro from murine prostate stem cells. Stem Cells. 2007;25:2760-2769.

5 Liu X, Chen X, Rycaj K et al. Systematic dissection of phenotypic, functional, and tumorigenic heterogeneity of human prostate cancer cells. Oncotarget. 2015;6:23959-23986.

6 Jeter CR, Badeaux M, Choy G et al. Functional evidence that the self-renewal gene NANOG regulates human tumor development. Stem Cells. 2009;27:993-1005.

7 Bhatia B, Jiang M, Suraneni M et al. Critical and distinct roles of p16 and telomerase in regulating the proliferative life span of normal human prostate epithelial progenitor cells. J Biol Chem. 2008;283:27957-27972.

8 Debacq-Chainiaux F, Erusalimsky JD, Campisi J et al. Protocols to detect senescence-associated beta-galactosidase (SA-betagal) activity, a biomarker of senescent cells in culture and in vivo. Nat Protoc. 2009;4:1798-1806.

9 Xin L, Ide H, Kim Y et al. In vivo regeneration of murine prostate from dissociated cell populations of postnatal epithelia and urogenital sinus mesenchyme. Proc Natl Acad Sci U S A. 2003;100 Suppl 1:11896-11903.

10. Zhang D, Park D, Zhong Y et al. Stem cell and neurogenic gene expression profiles link prostate basal cells to aggressive prostate cancer Nat Commun. 2016;In Press.

11 Wang Y, Wang Y, Liu Q et al. Comparative RNA-seq analysis reveals potential mechanisms mediating the conversion to androgen independence in an LNCaP progression cell model. Cancer Lett. 2014;342:130-138.

12 Liu P, Ramachandran S, Ali Seyed M et al. Sex-determining region Y box 4 is a transforming oncogene in human prostate cancer cells. Cancer Res. 2006;66:4011-4019.

13 Holzbeierlein J, Lal P, LaTulippe E et al. Gene expression analysis of human prostate carcinoma during hormonal therapy identifies androgen-responsive genes and mechanisms of therapy resistance. Am J Pathol. 2004;164:217-227.

14 Rajan P, Sudbery IM, Villasevil ME et al. Next-generation sequencing of advanced prostate cancer treated with androgen-deprivation therapy. Eur Urol. 2014;66:32-39.

15 Lehmusvaara S, Erkkila T, Urbanucci A et al. Chemical castration and anti-androgens induce differential gene expression in prostate cancer. J Pathol. 2012;227:336-345.

16 Tamura K, Furihata M, Tsunoda T et al. Molecular features of hormone-refractory prostate cancer cells by genome-wide gene expression profiles. Cancer Res. 2007;67:5117-5125.

17 Varambally S, Yu J, Laxman B et al. Integrative genomic and proteomic analysis of prostate cancer reveals signatures of metastatic progression. Cancer Cell. 2005;8:393-406.

18 Irshad S, Bansal M, Castillo-Martin M et al. A molecular signature predictive of indolent prostate cancer. Sci Transl Med. 2013;5:202ra122.

19 Beltran H, Rickman DS, Park K et al. Molecular characterization of neuroendocrine prostate cancer and identification of new drug targets. Cancer Discov. 2011;1:487-495.

20 Tzelepi V, Zhang J, Lu JF et al. Modeling a lethal prostate cancer variant with small-cell carcinoma features. Clin Cancer Res. 2012;18:666-677.

21 Lapuk AV, Wu C, Wyatt AW et al. From sequence to molecular pathology, and a mechanism driving the neuroendocrine phenotype in prostate cancer. J Pathol. 2012;227:286-297.

22 Tibshirani R, Hastie T, Narasimhan B et al. Diagnosis of multiple cancer types by shrunken centroids of gene expression. Proc Natl Acad Sci U S A. 2002;99:6567-6572.

23 Yamamoto H, Masters JR, Dasgupta P et al. CD49f is an efficient marker of monolayer- and spheroid colony-forming cells of the benign and malignant human prostate. PLoS One. 2012;7:e46979.

24 Hoogland AM, Verhoef EI, Roobol MJ et al. Validation of stem cell markers in clinical prostate cancer: alpha6-integrin is predictive for non-aggressive disease. Prostate. 2014;74:488-496.

Supplementary Figures

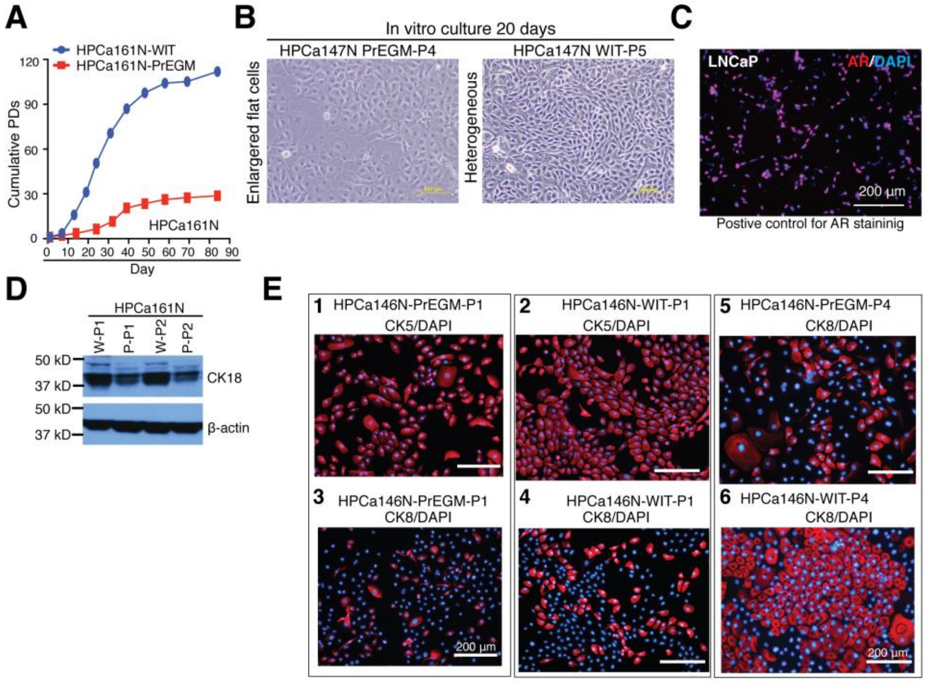

Figure S1. Faster proliferation and extended lifespan of HPE cells cultured in WIT compared to PrEGM.

(A) Cumulative PDs of primary benign human prostate bulk epithelial cells (purified from HPCa161) cultured in either PrEGM or WIT medium.

(B) Phase contrast images of the indicated cultures showing the relatively homogeneous and heterogeneous morphologies displayed by prostate epithelial cells cultured in PrEGM and WIT, respectively. Note that the PrEGM cultures at P4 were largely senescent (large and flat) whereas the WIT cultures at P5 were still vigorously proliferating.

(C) IF of AR in LNCaP cells showing the reliability of the antibody used in Figure 2F.

(D) Western blot analysis of CK18 expression in prostate epithelial cells freshly purified from HPCa116 and cultured in WIT (W) or PrEGM (P) for 2 different passages (P1 and P2). CK18 was detected as a major ~40 kD band and a minor 48 kD band.

(E) IF of CK5 and CK8 in human benign primary prostate epithelial cells from HPCa146N cultured in PrEGM and WIT at different passages (P). Note that although ~100% cells at P1 in both PrGEM and WIT cultures were $CK5^+$ (compare panels 1 & 2), the WIT cultures at both P1 (compare panels 3 and 4) and P4 (panels 5 vs. 6) showed significantly more $CK8^+$ cells than corresponding PrEGM cultures.

Scale bars (B, C, E), 200 µm.

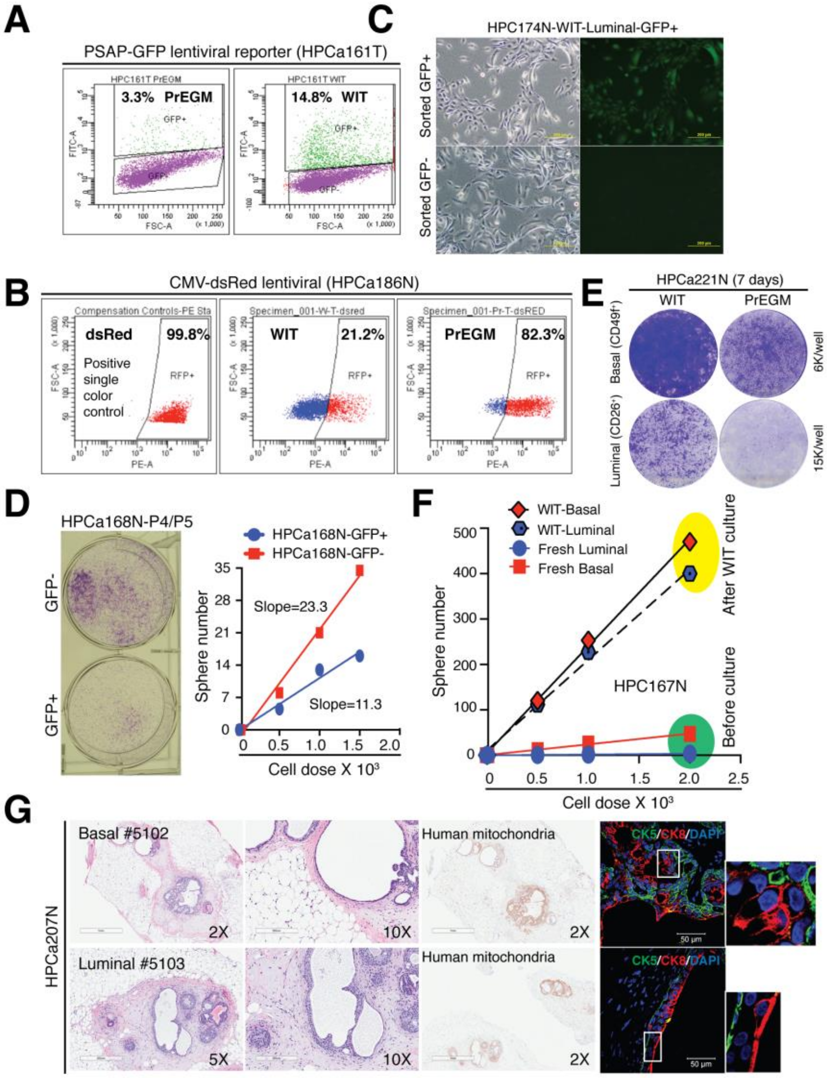

Figure S2. WIT supports the growth of luminal progenitor cells.

(A) FACS analysis of % GFP$^+$ cells in PSAP-GFP lentivirus infected HPE cells originally cultured in PrEGM or WIT.

(B) FACS analysis of % RFP$^+$ cells in CMV-dsRed lentivirus infected cells originally cultured in either PrEGM or WIT.

(C) GFP$^+$ (PSA$^+$) and GFP$^-$ (PSA$^-$) cells remain unchanged in WIT in both the presence and absence (not shown) of DHT (10 nM). Image magnification, 10X.

(D) Colony formation (left) and sphere formation (right) assays showing PSA$^+$ cells exhibiting lower stem/progenitor activities than PSA$^-$ cells.

(E) WIT represents a better system for propagating both human primary basal and luminal cells. Shown are colony formation images of freshly purified basal (CD26$^-$CD49f$^+$) and luminal (CD26$^+$CD49f$^-$) cells grown in the two media.

(F) Limiting dilution sphere assays of freshly purified human prostatic basal and luminal cells (before culture, green) and cells after a primary expansion in WIT (after culture, yellow).

(G) H&E, human-specific mitochondria staining, and IF analysis of CK5 and CK8 in prostate tissues regenerated *in vivo* from primary WIT-cultured HPCa207N basal and luminal cell populations. Scale bars, 1 mm (2X images), 400 µm (5X images), 200 µm (10X images), and 50 µm (confocal images).

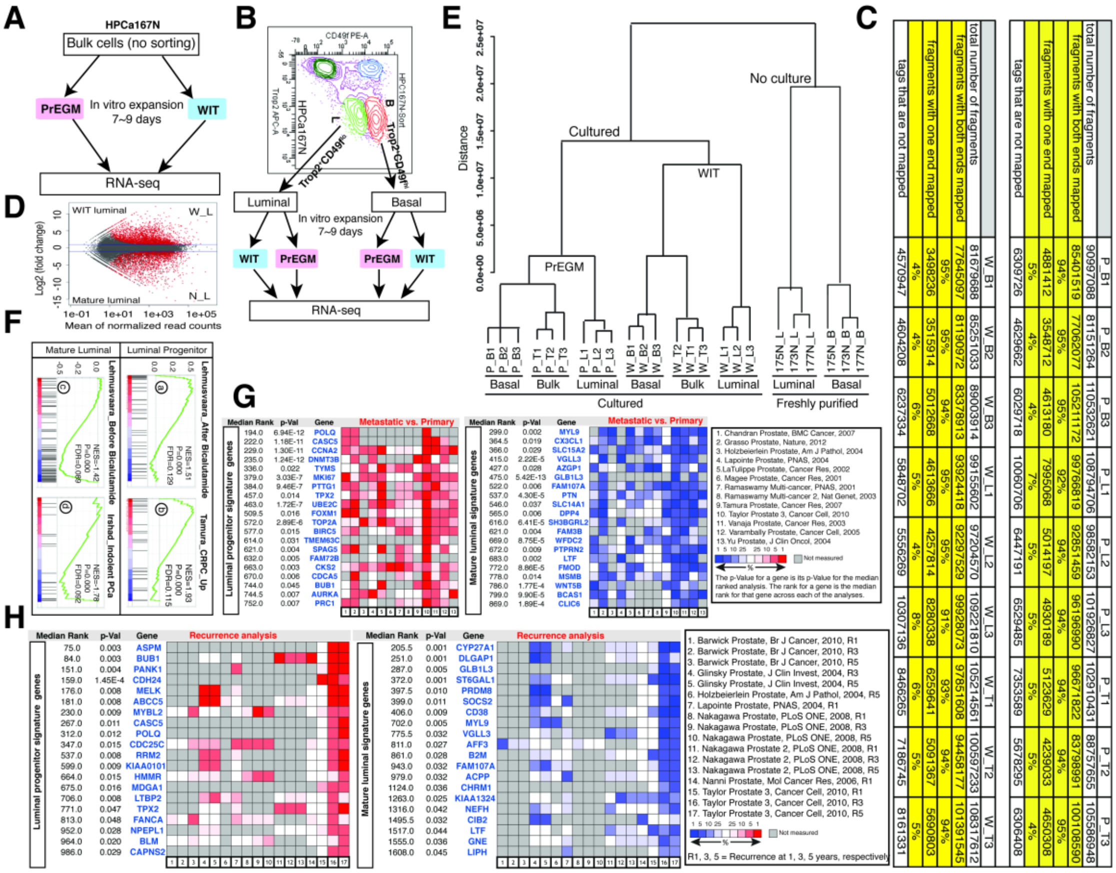

Figure S3. Distinct transcriptomes of mature basal cells and luminal cells, basal SCs, and luminal progenitors.

(A, B) Schematic of RNA-Seq experiments on cultured primary human prostate epithelial cells. Note that each cell sample has 3 technical repeats for RNA-Seq experiments.

(C) Alignment results showing the high quality of our RNA-Seq data evidenced by high mapping rate.

(D) The MA plot showing the appropriate normalization of our RNA-Seq data. The red dots represent the genes with p value <0.05 (i.e., DEGs).

(E) Unsupervised hierarchical clustering of RNA-Seq data from the indicated cell cultures and populations. P_T, P_B and P_L, disassociated human benign prostate total (T, bulk) cells, FACS-purified basal (B) and luminal (L) cell populations cultured in PrEGM (P). W_T, W_B and W_L, disassociated total (bulk) cells, FACS-purified basal and luminal cell populations cultured in WIT (W). N_B and N_L, 3 pairs of freshly purified benign prostatic mature basal and luminal cells. Y-axis shows euclidean distance for log2 (normalized read counts).

(F) GSEA showing for the enrichment of indicated gene signatures in luminal progenitor cells (W_L) compared to freshly purified mature luminal cells (N_L).

(G,H) Oncomine concept analysis of the indicated two gene sets showing that many of the luminal progenitor genes and mature luminal genes are positively and negatively associated with metastasis and tumor recurrence, respectively. Shown are the top 20 genes for each category.

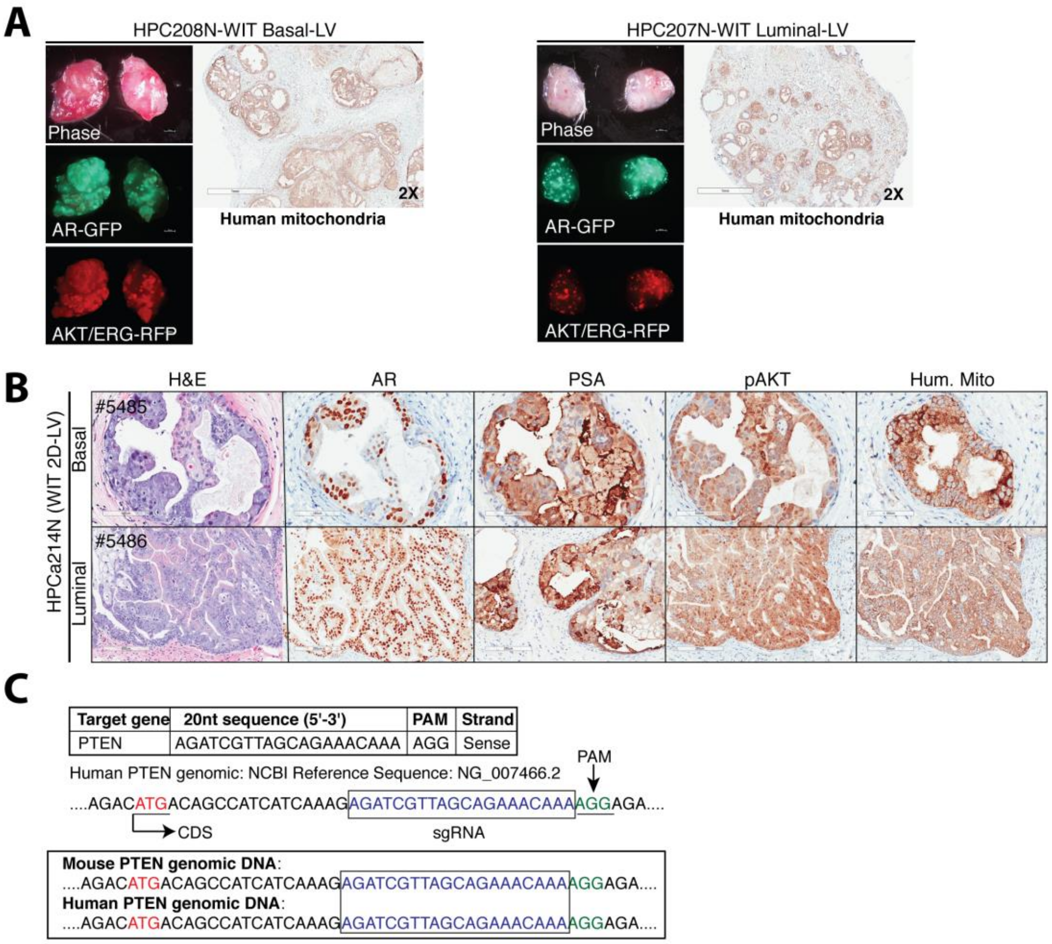

Figure S4. Both culture-enriched basal SCs and luminal progenitors can be oncogenically transformed to initiate human PCa.

(A) Fluorescent images showing that the endpoint tumors were originated from human prostatic cells infected with lentivirus, as validated by human-specific mitochondria staining. Scale bars, 1mm.

(B) H&E and IHC analysis of the indicated genes in tumors derived from the indicated cell types. Scale bars, 100 µm (top) and 200 µm(bottom).

(C) Schematic of the sequence of an sgRNA targeting mouse and human PTEN. Note that the sequences for human and mouse genomic PTEN covering the sgRNA region are the same.

Supplemental Table

Table S1. Primary benign human prostate tissue samples used in the current study*

HPC #	Date	Age	GS	Experiments	Related Figures
HPCa146N	9/20/12	56	7	Cell culture, PDs, Sphere formation,	Fig. 2A-2B, 2D-2E
HPCa147N	9/24/12	67	7	Cell culture, PDs, SA-β-gal staining,	Fig. 2C, S1C, Repeat e
HPCa151N	11/5/12	67	8	Cell culture, Colony formation	Fig 2H
HPCa152N	11/5/12	50	7	Cell culture, Colony formation	Fig 2H
HPCa154N	11/19/12	63	9	Cell culture, PSAP-GFP-LV, FACS, qRT-PCR	Fig. 3D
HPCa159N	1/7/13	50	6	Cell culture, qRT-PCR, IF (CK19)	Fig. 3I-3K
HPCa161N	1/17/13	74	9	Cell culture, PDs, SA-β-gal staining, Western Blot, PSAP-GFP-LV, FACS	Fig. 2C, 2G, 3B, 3E-3F,
HPCa161T	1/17/13	74	9	Cell culture, PSAP-GFP-LV, FACS	Fig. S2A
HPCa167N	3/13/13	67	6	RNA-Seq, Cell culture, FACS, sphere formation	Fig. 3H, S3A-3E, S2E
HPCa168N	4/1/13	76	7	Cell culture, qRT-PCR, ALDH analysis, PSAP-GFP-LV, FACS	Fig. 2I-2J, 3A, S2D
HPCa173N	6/24/13	62	7	RNA-Seq (Zhang et al., 2015)	Fig. 4D-4G, S3D (used
HPCa175N	7/15/13	67	7	RNA-Seq, (Zhang et al., 2015)	Fig. 4D-4G, S3D (used
HPCa177N	7/29/13	74	6	RNA-Seq, (Zhang et al., 2015)	Fig. 4D-4G, S3D (used
HPCa174N	7/8/13	69	7	Cell culture, PDs, Sphere formation,	Fig. 3C, 3L, S2C
HPCa179N	8/19/13	68	6	Cell culture, TR, IF (AR)	Fig. 2F, 3M
HPCa186N	12/2/13	65	6~7	Cell culture, colony/sphere formation, CMV-dsRED-LV, FACS	Fig. 3G, S2B
HPCa207N	10/27/14	59	7~8	Cell culture, TR assays, Cell transformation assays	Fig. 5B, S2F, S4A
HPCa208N	11/3/14	72	7~8	Cell culture, Organoid culture, TR assays, Cell transformation (TR) assays	Fig. 4B, 5D, 1C, S4A
HPCa214N	3/2/15	59	6~7	Cell culture, Organoid culture, Cell transformation (TR) assays	Fig. S4B, 1A
HPCa221N	7/20/15	71	8	FACS (CD26/CD49f), Cell culture, Colony formation, Organoid culture	Fig. S2E, Repeat exp. I
HPCa224N	9/28/15	64	6~7	FACS (CD26/CD49f), Cell culture, Colony formation, Organoid culture	Repeat exp. For Fig. 1
HPCa195N	3/31/14	63	6~7	Cell culture, Cell transformation (TR) assays	Repeat exp. For Fig. 5
HPCa202N	7/7/14	61	7	Cell culture, Cell transformation (TR) assays	Repeat exp. For Fig. 5
HPCa204N	9/8/14	68	7	Cell culture, Cell transformation (TR) assays	Repeat exp. For Fig. 5
HPCa215N	3/9/15	68	6~7	Cell culture, Cell transformation (TR) assays, Organoid culture	Repeat exp. For Fig. 5 exp. For Fig. 1A (data
HPCa217N	5/4/15	53	7	Cell culture, Cell transformation (TR) assays	Repeat exp. For Fig. 5
HPCa219N	6/15/15	59	7	Cell culture, Cell transformation (TR) assays, Organoid culture	Repeat exp. For Fig. 5 Repeat exp. For Fig. 1

*A total of 27 patient derived benign prostate tissues were used in this study. These benign tissues were based on negative biopsies. The exact utilization of each sample in different figures is also labeled in the

panels or indicated in the figure legends. Bulk culture means the cultures were directly established by plating the single cell suspensions from dissociated benign tissues. Normally, prostatic epithelial cells were freshly FACS-purified from dissociated tissues according to the expression of CD49f/Trop2 and CD26/CD49f, and were then used in various functional studies including proliferation, clonal, sphere, tissue recombination assays and oncogene-encoding lentivirus-mediated cell transformation assays. GS, gleason score; PDs, population doubling experiments (cell growth kinetic); LV, lentivirus treatment.
